# The Microstructure and Mechanical Properties of Multi-Strand, Composite Welding-Wire Welded Joints of High Nitrogen Austenitic Stainless Steel

**DOI:** 10.3390/ma12182944

**Published:** 2019-09-11

**Authors:** Jianguo Li, Huan Li, Yu Liang, Pingli Liu, Lijun Yang

**Affiliations:** 1 School of Materials Science and Engineering, Tianjin University, Tianjin 300072, China; 2 Tianjin Key Laboratory of Advanced Joining Technology, Tianjin 300072, China; 3 Jiangsu Lianjie Welding Technology Co., Jiangyin 214400, China

**Keywords:** high nitrogen austenitic stainless steel, multi-strand composite welding wire, electron backscatter diffraction, welding thermal cycle, mechanical property, microstructure

## Abstract

A multi-strand composite welding wire was applied to join high nitrogen austenitic stainless steel, and microstructures and mechanical properties were investigated. The electrical signals demonstrate that the welding process using a multi-strand composite welding wire is highly stable. The welded joints are composed of columnar austenite and dendritic ferrite and welded joints obtained under high heat input and cooling rate have a noticeable coarse-grained heat-affected zone and larger columnar austenite in weld seam. Compared with welded joints obtained under the high heat input and cooling rate, welded joints have the higher fractions of deformed grains, high angle grain boundaries, Schmid factor, and lower dislocation density under the low heat input and cooling rate, which indicate a lower tensile strength and higher yield strength. The rotated Goss (G_RD_) ({110}〈1$\bar{1}$0〉) orientation of a thin plate and the cube (C) ({001}〈100〉) orientation of a thick plate are obvious after welding, but the S ({123}〈63$\bar{4}$〉) orientation at 65° sections of Euler’s space is weak. The δ-ferrite was studied based on the primary ferrite solidification mode. It was observed that low heat input and a high cooling rate results in an increase of δ-ferrite, and a high dislocation density was obtained in grain boundaries of δ-ferrite. M_23_C_6_ precipitates due to a low cooling rate and heat input in the weld seam and deteriorates the elongation of welded joints. The engineering Stress–strain curves also show the low elongation and tensile strength of welded joints under low heat input and cooling rate, which is mainly caused by the high fraction of δ-ferrite and the precipitation of M_23_C_6_.

## 1. Introduction

High nitrogen austenitic stainless steel (HNASS) is a novel engineering material utilising nitrogen (>0.4%), which is used as a substitute for nickel in conventional high-nickel austenitic stainless steel. Nitrogen is a strengthening interstitial element which improves the mechanical strength and corrosion resistance of materials and enlarges the austenite range [1,2]. Additionally, nitrogen reduces allergic reactions and the high cost of conventional austenitic stainless steel due to nickel. Therefore, HNASS is widely used in power plants, shipbuilding, petroleum, pressure vessels, and the medical industry. However, the application of HNASS in structural components is limited by many welding issues. Kamiya et al. [3] conducted the gas tungsten arc welding (GTAW) experiments of austenitic stainless steel containing 0.51% N and 0.78% N to study the loss of nitrogen and reported the loss of nitrogen and porosities observed along the complete fusion boundary of the weld. Ogawa et al. [4] reported that nitride precipitates after aging 2 s in the temperature range between 900 °C and 1100 °C at the welding heat-affected zone (HAZ) of high nitrogen stainless steel containing about 1% nitrogen, and decreases the critical pitting corrosion temperature. Recently, Zhang et al. [5] studied the effect of brazing temperature on the microstructure and mechanical properties of HNASS joints brazed with Ni–Cr–P filler and also found Cr_2_N compounds at the HNASS/filler interface when the temperature was below 1000 °C. The solidification cracking due to nitrogen addition was also found by Woo and Kikuchi [6]. These phenomena greatly reduce the mechanical properties and corrosion resistance of welded joints.

To solve these issues, Hertzman [7] studied the effect of nitrogen on the microstructure and properties of austenitic stainless steel and observed that the nitrogen loss of weld pools was avoided by controlling the shielding gas nitrogen content in tungsten inert gas (TIG) welding. However, nitrogen addition results in the presence of nitrides. Hosseini et al. [8] also demonstrated that nitrogen loss with the increasing number of passes and heat input resulted in the formation of large ferrites and nitrides, and recommended using shielding gases containing-nitrogen and filler metals. Qiang and Wang [9] employed the double-sided synchronous autogenous GTAW and studied the effect of shielding gases on HNASS welding. However, this process is relatively unstable. Additionally, friction stir welding (FSW), which is a solid-state welding process, was first adopted by Park et al. [10] and obtained the same nitrogen content as the base metal. Nonetheless, the nitride was formed in the HAZ. Li et al. [11] obtained a higher yield strength and ultimate tensile strength than base metals in the welding process of HNASS using FSW, but the elongation was reduced significantly. Zhang et al. [12] studied the microstructure and corrosion resistance of a FSW high nitrogen stainless steel joint and obtained the heavily corroded, thermomechanically affected zone due to the high defect density, galvanic corrosion and the formation of Cr-rich particles. Shielded metal arc welding, GTAW, electron beam welding, and FSW processes were compared by Mohammed et al. [13], and improved mechanical properties were obtained in FSW compared to fusion welds. However, good support for workpieces is required in FSW and this process is not practical for component welding for complicated shapes. 

For applications, gas metal arc welding (GMAW) is the most convenient, reliable, and widely used welding technique. Cortes-Cervantes et al. [14] used GMAW to join super-austenitic stainless steel and studied the electromagnetic interactions. Toit [15] employed E307, ER2209, and 15CrMn to pair with Cromanite, and E307 produced sound weld joints with excellent mechanical properties. Mohammed et al. [16] used the commercially high strength fillers of precipitation hardenable (PH) 13-8 Mo filler and nickel based (MDN 250) 18Ni filler to weld high nitrogen stainless steel and found that the filler wire composition has a significant role on microstructure, mechanical properties, and corrosion behaviour. However, no matching filler metals can be used to join HNASS at present. A multi-strand composite welding wire is proposed as rotating wire to join HNASS in GMAW. Zhang et al. [17] also reported that the rotating wire GMAW process changes the fluid flow of molten pools, refined microstructure, and markedly improves the tensile strength. Moreover, for high nitrogen stainless steel, microstructure and mechanical properties of welded joints are sensitive to heat input and cooling rate during welding. Hence, the study on microstructure and mechanical properties of welded joints combined under the different welding thermal cycles by the multi-strand composite welding wire is very necessary. Electron backscatter diffraction (EBSD) is a promising technique which is widely used by material researchers, scientists, and engineers. Randle [18] demonstrated that this technique can measure microtexture and microstructure fractions, characterise the grain and phase boundaries, identify phases, and determine the strain. Furthermore, Zhang et al. [19] also evaluated the plastic deformation by kernel average misorientation (KAM) of EBSD analyses technique and Roach et al. [20] demonstrated the preferential sites of fatigue crack using the Schmid factor of EBSD.

Herein, HNASSs were joined under the different thermal cycles by multi-strand composite welding wire GMAW. The microstructures and local plastic strain of weld joints were estimated by measuring the dislocation densities, misorientation angles, recrystallised grains, Schmid factors, and crystal orientations via EBSD. The mechanical properties were measured by a tensile test, and the fracture micrographs were observed. To further study the strengthening mechanism, microstructures of weld seam were investigated.

## 2. Experimental Procedures

**Figure 1:**
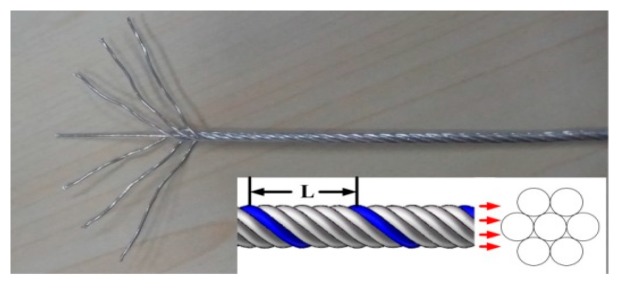
Schematic of welding wire.

HNASS plates with different dimensions of 300 mm × 150 mm × 14 mm and 300 mm × 150 mm × 8 mm were applied. HNASS was joined by a multi-strand composite welding wire called TP-N1670. The welded joint with 8 mm thickness of base metal is called 1670-8, and the other is called 1670-14. The welding wires TP-N1670 were provided by Jiangsu Lianjie Welding Technology Co., Ltd, Jiangyin, China. The maximum diameter of the welding wire was 1.6 mm. The welding wire, as illustrated in Figure 1, was composed of seven wires with a diameter of 0.53 mm, and the equivalent diameter was 1.4 mm. In Figure 1, L represents the feeding length when welding wire rotated a cycle, and for TP-N1670, L measured 12.5 mm. The chemical compositions of the HNASS and the welding wire are listed in Table 1.

**Figure 2:**
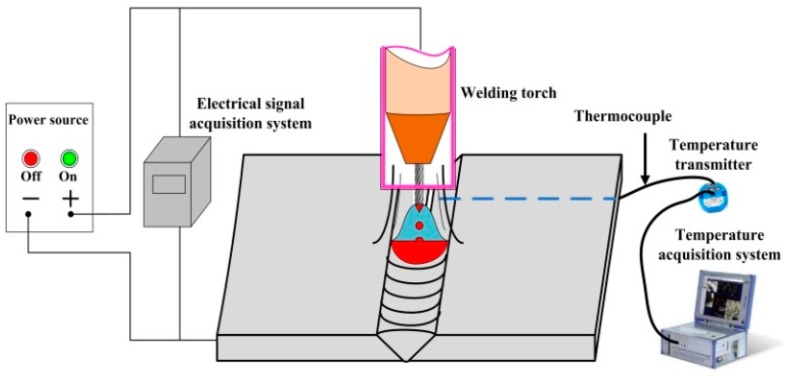
Schematic diagram of the welding trials.

Welding trials for the different HNASSs were conducted. The equipment system for the welding trials is presented in Figure 2. The main system components consisted of a power source, welding torch, electrical signal acquisition system, thermocouple, and temperature acquisition system. The EWM welding power source was operated in direct current electrode positive (DCEP) condition, and a shielding gas of 97.5% Ar + 2.5% CO_2_ was used in the welding process. The welding parameters for HNASSs were kept consistent, as presented in Table 2. The current and voltage waveforms were obtained by using an electrical signal acquisition system with a sampling rate of 100 kHz. Moreover, a DEWETRON (Graz, Austria) temperature acquisition system was used to obtain the welding thermal cycle curves. The type K thermocouples with a range from 0 to 1100 °C were glued to the bottom surface using aluminium tape, and the distance from the weld seam centre was 6 mm. 

**Figure 3:**
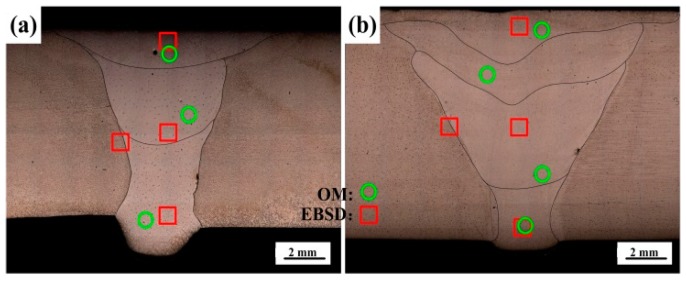
Transverse section of the welded joints for the (**a**) 1670-8 and (**b**) 1670-14.

After welding, three tensile test samples for 1670-8 and 1670-14 were cut perpendicularly to the welded joints, respectively. The tensile tests were conducted using an electromechanical universal testing machine (CRIMS DDL300, Changchun, China) at a strain rate of 0.5 s^−1^, and the fracture surfaces were observed using scanning electron microscopy (SEM; ZEISS Supra 55, Jena, Germany). The transverse sections of the welded joints with different thicknesses are presented in Figure 3. It is observed that the welded joints of 1670-8 and the 1670-14 are composed of three passes and four passes, respectively. The chemical compositions of weld seam were measured by X-ray fluorescence spectrograph (Axios, Panako, Almelo, The Netherlands), and nitrogen contents were measured by Oxygen and Nitrogen Analyzer (EMGA-820, HORIBA, Kyoto, Japan), as shown in Table 1. The microstructures of the welded joints were characterised with optical microscopy (OM, OLYMPUS BX51M, Olympus, Tokyo, Japan) and the EBSD (SYMMETRY, Oxford Instruments, Abingdon, UK) system equipped with an SEM. The locations observed were marked in Figure 3, and the red boxes indicate the location of the EBSD study and green cycles indicate the OM study. The OM samples were machined from the welded joints, ground, mechanically polished, and etched in a mixed reagent of HCl and HNO_3_ (3:1) for 30 s. The EBSD samples were prepared by grinding, mechanically polishing, and electrolytically polishing in a reagent of ethyl alcohol and perchloric acid (15:85, volume fraction) at 20 V for 15 s. EBSD was carried out using in a fine step size of 5 μm and a scanning area of 231 × 173 μm^2^ and a fine step size of 0.2 μm and a scanning area of 80 × 80 μm^2^. For the identification of precipitates and the investigation of a deformation mechanism, transmission electron microscopy (TEM; JEM-2100, JEOL, Tokyo, Japan) was applied.

## 3. Results and Discussion

### 3.1. Welding Thermal Cycle and Electrical Signal

**Figure 4:**
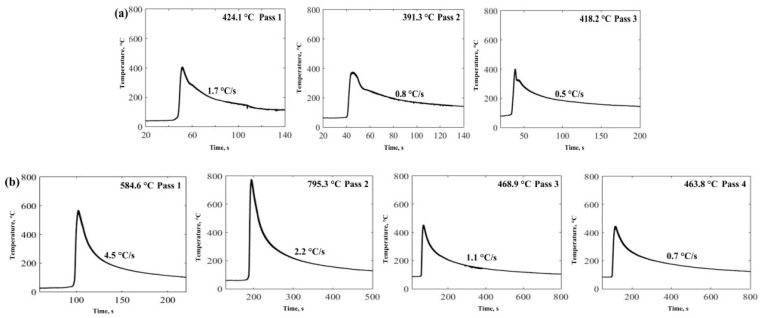
Thermal cycle curves for each pass during welding for the (**a**) 1670-8 and (**b**) 1670-14.

**Figure 5:**
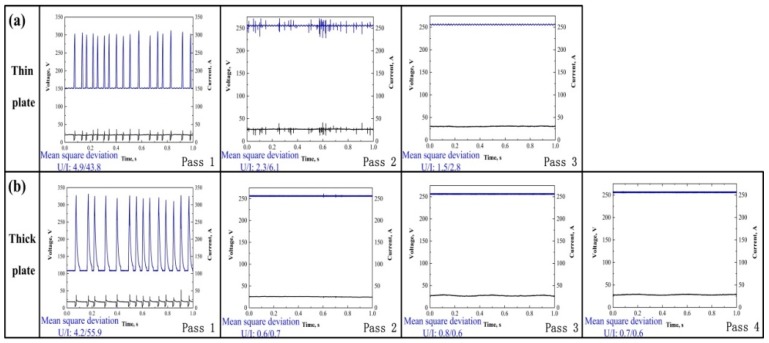
Electrical signal oscillograms for the (**a**) 1670-8 and (**b**) 1670-14. U/I represents the square deviation value of the thin and thick plates in 1 s.

The HNASS plates with different thicknesses were joined by GMAW using a multi-strand composite welding wire. The thermal cycle curves measured clearly illustrate the change in heat input and cooling rate during welding. Table 2 displays a higher heat input for 1670-14. Figure 4 presents the thermal cycle curves at 6 mm from the welding seam centre. According to Figure 4a, the peak temperature of 1670-14 at every pass is higher than that of 1670-8 in the welding process. Moreover, as observed in Figure 4b, 1670-14 has a higher average cooling rate than 1670-8 at the first pass, and the average cooling rate of 1670-14 decreases with the increase of the current and voltage. Figure 5 shows the current waveforms, voltage waveforms. The current and voltage waveforms at pass 1 reflect a highly stable short-circuiting transfer process, and a high short-circuiting transfer frequency reveals the stability and homogeneity of the droplet transfer. The low mean square deviation also reflects a stable welding droplet transfer process. During the welding, when the arc shortens, the voltage value decreases. The voltage and current waveforms of 1670-8 and 1670-14 at other passes have little fluctuation, which indicates that the modes of the droplet transfer represent the spray transfer, and the stability is higher than that of short-circuiting transfers. Moreover, it is observed that the welding process of 1670-14 is more stable than that of 1670-8.

### 3.2. Microstructures of the Welded Joints

**Figure 6:**
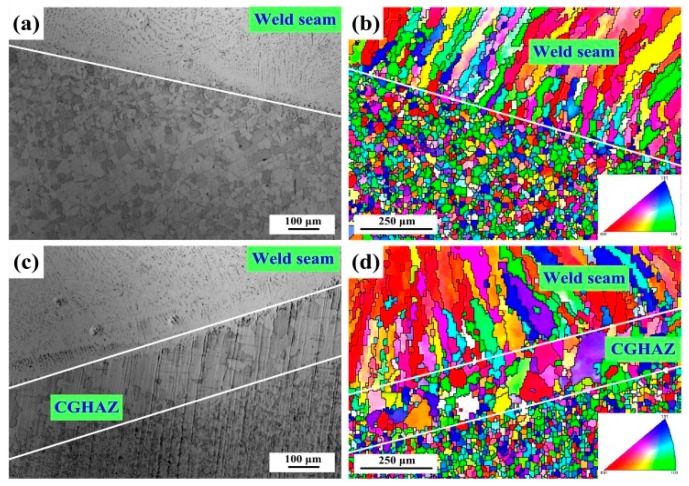
Microstructures (**a**) observed by OM and (**b**) an inverse pole figure (IPF) for 1670-8; and (**c**) observed by OM and (**d**) an IPF for 1670-14.

The OM and EBSD maps of the welded joints are observed in Figure 6. The base metal is composed of equiaxed austenite, and the weld seam is composed of columnar austenite and dendritic δ-ferrite. Following the welding thermal cycles, the weld seam produces a preferential growth of columnar grains. The grains of the base metal in the vicinity of the fusion line are affected, and the recovery and recrystallisation occur. High heat input is in favour of the growth of columnar grains and the formation of a coarse-grained heat-affected zone (CGHAZ). Therefore, 1670-14 has larger columnar grains, and the CGHAZ is produced due to the local high heat input of the base metal. The maximum width of the CGHAZ is 240 μm. As 1670-8 has low heat input, the CGHAZ is not formed. 

**Figure 7:**
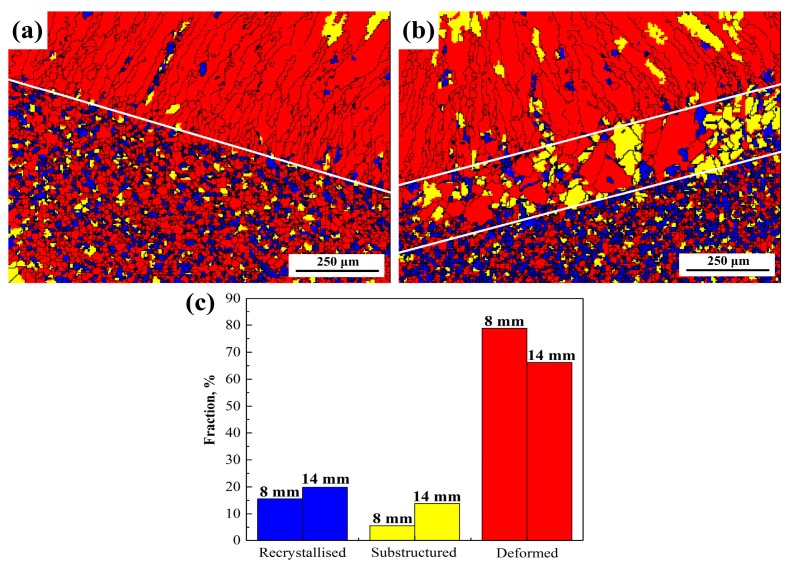
Recrystallised grain distribution diagram for (**a**) 1670-8 and (**b**) 1670-14, and (**c**) the fraction of grains for the different thicknesses of welded joints.

The grains of the welded joints change after the welding. When the average angle of a grain is greater than 3°, the grain is called a deformed grain. When the average angle is smaller than 3°, and the misorientation angle between grains is greater than 3°, the grain is called subgrains. The interface between adjoining grains is called sub-boundaries. The remaining grains are called recrystallised grains. From Figure 7a,b, it can be observed that the deformed grains gather in the weld seam, and the weld seam has few subgrains and recrystallised grains. The subgrain fraction of 1670-14 is higher than that of 1670-8 in the weld seam. Moreover, the recrystallised grains form along the deformed grains, which mainly results from the high driving force of nucleation in the deformed grains. The CGHAZ of 1670-14 is mainly composed of subgrains and deformed grains. According to Figure 7c, the percentages of recrystallised grains, subgrains, and deformed grains for 1670-14 is 20%, 14%, and 66%, respectively; while 1670-8 due to the lower heat input and the lesser number of passes, presents a higher percentage of deformed grains (79%) and a lower percentage of subgrains (5%). In fact, the fraction of sub-boundaries rises with the increase of subgrains. Lu et al. [21] reported that the high sub-boundary fraction reflects the high dislocation density.

**Figure 8:**
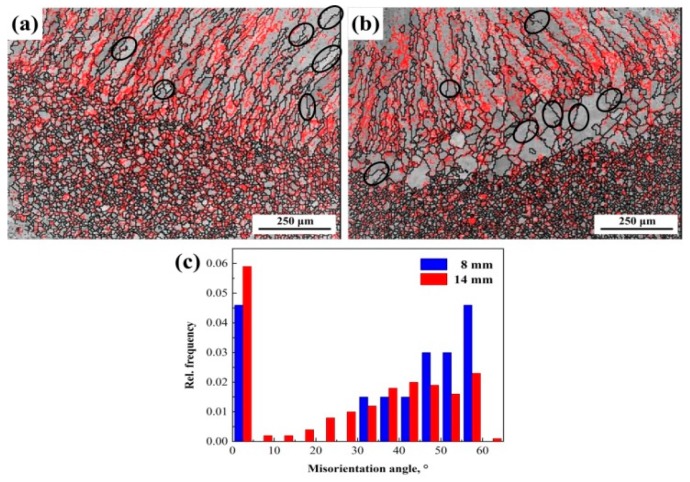
Grain boundary maps for the (**a**) 1670-8, (**b**) 1670-14, and (**c**) the relative frequency of misorientation angle.

Figure 8a,b represents the grain boundary distributions, and red line describes the low angle grain boundaries (LAGBs) (2°–15°). The black line describes the high angle grain boundaries (HAGBs) (>15°). Figure 8c shows the relative frequency (Rel. frequency) of misorientation angle. The welded joint of 1670-14 presents a higher percentage of LAGBs in the selective area (32.4%) than that of 1670-8. The regions with a high percentage of LAGBs indicate a high degree of sensitization to concentration of dislocation density. Parts of HAGBs are marked in the black cycle. From Figure 7 and Figure 8, it can be deduced that most of the recrystallised grains are formed along the HAGBs. Mccabe et al. [22] also reported that recrystallisation occurs because of the motion of HAGBs and the nucleation is preferentially formed close to HAGBs. High heat input and number of welding passes improve the formation of recrystallized grains along HAGB.

**Figure 9:**
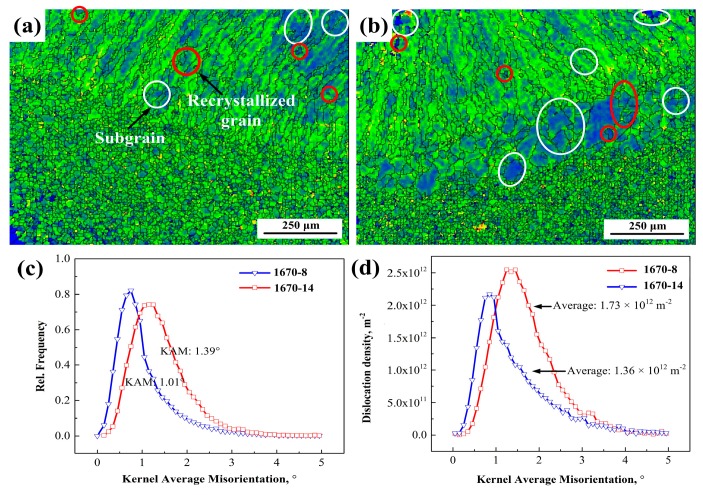
(**a**) Kernel average misorientation (KAM) map of 1670-8, (**b**) KAM map of 1670-14, (**c**) KAM distribution, and (**d**) dislocation density.

Owing to the preferential growth of grains in the weld seam, and grain coarsening in the HAZ, parts of the neighbouring grains deform plastically during the welding, leading to the inhomogeneous local misorientation. Kamaya [23] reported that the local misorientation was consistent with the geometrically necessary dislocation density. The KAM represents the average misorientation between neighbouring points. KAM maps reflect the distribution of the dislocation density. The measurement conditions and step size have a considerable influence on KAM maps, and all the measurements were conducted under the same conditions, with a step size of 5 μm. The KAM maps of 1670-8 and 1670-14 are presented in Figure 9a,b. The dislocation density of 1670-14 is higher than that of 1670-8 in the weld seam. A sparse dislocation region is formed in the CGHAZ of 1670-14. As stated above, dislocations are mainly concentrated in deformed grains and sub-boundaries, and the dislocation density is low in subgrains and recrystallised grains, as observed in Figure 9a,b. The recrystallisation occurs easily in high dislocation density regions. The dislocation density decreases when the recrystallisation nucleation begins along deformed grains. Recrystallised grains form from deformed grains, which leads to the decrease of the dislocation density around recrystallised grains. Additionally, the HAGBs reduce the dislocation density, as observed in Figure 8 and Figure 9. Xu et al. [24] reported that the dislocation density was calculated as follows:

(1) $$\rho =\frac{2\theta }{\mu b}$$

where *ρ* is the dislocation density of a point, *θ* is the local misorientation angle, *μ* is the unit length of the point, and *b* is the Burgers vector. Figure 9c displays the KAM distribution in the selective area, and the average KAM values of 1670-8 and 1670-14 calculated are 1.01° and 1.39°, respectively. 1670-14 presents a larger KAM value than 1670-8, which indicates a higher dislocation density for 1670-14. Figure 9d presents the dislocation density calculated according to the Equation (1) and the average dislocation densities of 1670-8 and 1670-14 are 1.36 × 10^12^ m^−2^ and 1.73 × 10^12^ m^−2^, respectively. 1670-14 has a higher dislocation density than 1670-8. A high dislocation density leads to a large stress concentration with the increase of external force, and the plastic deformation has to overcome the considerable resistance of the dislocation accumulation. Moreover, the weld seam is the weakest zone for HNASS, and determines the mechanical properties of welded joint. Therefore, high fractions of LAGBs and dislocation density result in the stress concentration of 1670-14, which indicates that its welded joint has a better tensile strength than that of 1670-8 during deformation. 

**Figure 10:**
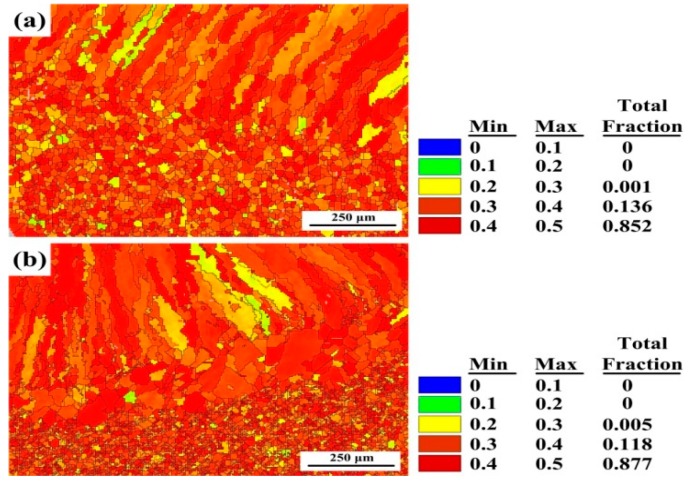
Schmid factor of the different thicknesses of plates along the fusion line: (**a**) 1670-8 and (**b**) 1670-14.

The Schmid factors of welded joints calculated through the EBSD process are presented in Figure 10. Gussevet al. [25] reported that the Schmid factor of soft grains is greater than 0.4, and that of hard grains is smaller than 0.35. Grains having a high Schmid factor are easier to slip with. The soft grain fractions of 1670-8 and 1670-14 are 85.2% and 87.7%, respectively. It can be observed that 1670-14 has a higher Schmid factor, and the grains oriented close to 〈101〉 have the lowest Schmid factor, as shown in Figure 6 and Figure 10. Hence, the plastic deformation is easier for 1670-14. In other words, 1670-14 has a lower yield strength than 1670-8. Furthermore, Ganesan et al. [26] studied the influence of nitrogen on properties of 316LN SS and demonstrated that a decrease of ferrite reduces the yield strength. During welding, low heat input and a high cooling rate increases the fraction of ferrite which has a considerable influence on yield strength.

**Figure 11:**
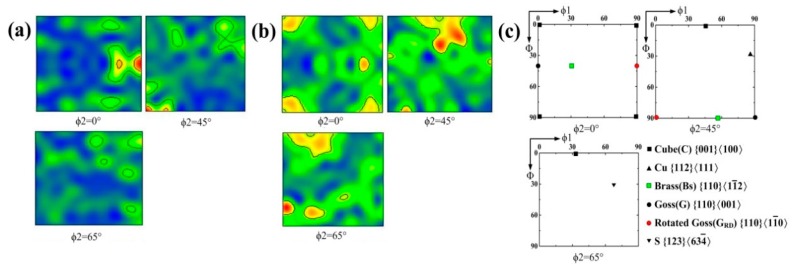
Orientation distribution function sections of Euler’s space for the (**a**) 1670-8, (**b**) 1670-14, and (**c**) standard orientation distribution functions (ODFs) for FCC material.

The crystallographic texture in metallic materials mainly results from the plastic deformation and the recrystallization of the metal. The S texture, brass (Bs) texture, and Cu texture originate from the plastic deformation, but the formation of Goss (G), rotated Goss (G_RD_) and cube (C) textures have a relationship with recrystallization. Figure 11 presents the orientation distribution functions (ODFs) of the weld joints and the standard ODF for face-centered cubic (FCC) materials at ϕ2 = 0°, 45°, and 65° sections of Euler’s space. In contrast with a standard ODF, the ODF of 1670-8 at ϕ2 = 0°, 45° clearly shows a typical rotated G_RD_ ({110}〈1$\bar{1}$0〉) orientation, whereas the ODF of 1670-14 at the same Euler’s space presents the cube C ({001}〈100〉) orientation. All changes in grain orientation are probably due to the different thermal cycle. The thermal cycle also weakens the intensity of the S ({123}〈63$\bar{4}$〉) orientation at 65° sections of Euler’s space, which indicates that rotated G_RD_ ({110}〈1$\bar{1}$0〉) texture and C ({001}〈100〉) texture form along an S ({123}〈63$\bar{4}$〉) orientation and weaken the S ({123}〈63$\bar{4}$〉) texture. What is more, S ({123}〈63$\bar{4}$〉) orientation can inhibit the crack propagation, and weak S ({123}〈63$\bar{4}$〉) orientation results in decreasing mechanical properties of welded joints.

### 3.3. Microstructures of the Weld Seam

**Figure 12:**
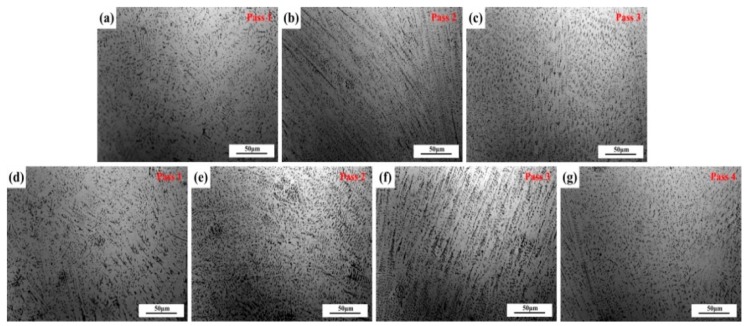
Weld seam microstructures of plates of different thicknesses at each pass, (**a**–**c**) for 1670-8 and (**d**–**g**) for 1670-14.

After welding using the multi-strand composite welding wire, the microstructures in the weld seam are different from those of the base metal, which depends on the solidification modes. Hammar and Svensson [27] established a relationship between the composition and solidification mode using Cr_eq_/Ni_eq_: Ni_eq_=Ni + 0.31Mn + 22C + 14.2N + Cu (wt.%) and Cr_eq_ = Cr + 1.37Mo + 3Ti + 1.5Si + 2Nb (wt.%). The values of Cr_eq_/Ni_eq_ for 1670-8 and 1670-14 are 1.53 and 1.61, respectively. Vasudevan et al. [28] reported that the primary ferrite mode C_req_/Ni_eq_ ratio is greater than 1.5. Primary ferrite has a considerable influence on the properties of welded joints. Bhaduri et al. [29] reported that low ferrite welded joints with Cr_eq_/Ni_eq_ < 1.5 were susceptible to hot-cracking. Lo et al. [30] reported that an austenite matrix containing ferrite presents favourable properties. Figure 12 displays the OM of the weld seam at every pass. Figure 12a–c represents the OM study of 1670-8 from bottom to top, and Figure 12d–g represents that of 1670-14. It can be observed that the growth direction of dendritic δ-ferrite is affected by the temperature gradient direction of solidification. Jahanzed et al. [31] reported that the directionality of δ-ferrite reveals the low-temperature gradient and cooling rate in this region. The cooling rate reduces with increasing weld passes. 

**Figure 13:**
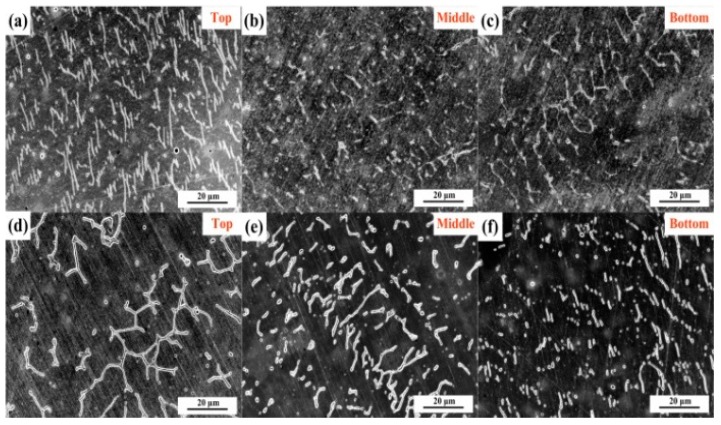
SEM images of weld seams at different places, (**a**–**c**) for 1670-8 and (**d**–**f**) for 1670-14.

The effect of welding thermal cycles on microstructure was further studied, and amplified microstructures of weld seams from top to bottom were obtained in Figure 13. According to the aforementioned, solidification of the weld seam occurs in primary the ferrite mode. When the cooling rate is high, the transformation of primary δ-ferrite into austenite is inhibited. Furthermore, thermal cycles of other passes can improve the transformation of δ-ferrite in pass one, and have no effect on the top. For the top of welded joints, 1670-8 has the low heat input and cooling rate. However, the top of 1670-8 has a higher fraction of δ-ferrite, which indicates that the fraction of δ-ferrite increases with the decreasing of the heat input. For the bottom of welded joints, the highest cooling rate and the lowest heat input should produce the highest fraction of δ-ferrite, but thermal cycles in other passes improve the change from network δ-ferrite to rod and granular δ-ferrite, as shown in Figure 13. 

**Figure 14:**
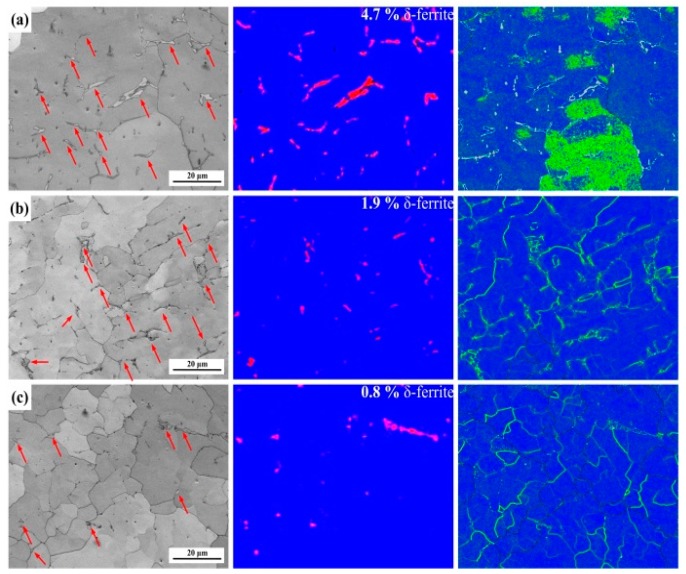
Contrast maps, phase distribution maps, and KAMs of 1670-8 at different places: (**a**) top, (**b**) middle, and (**c**) bottom.

**Figure 15:**
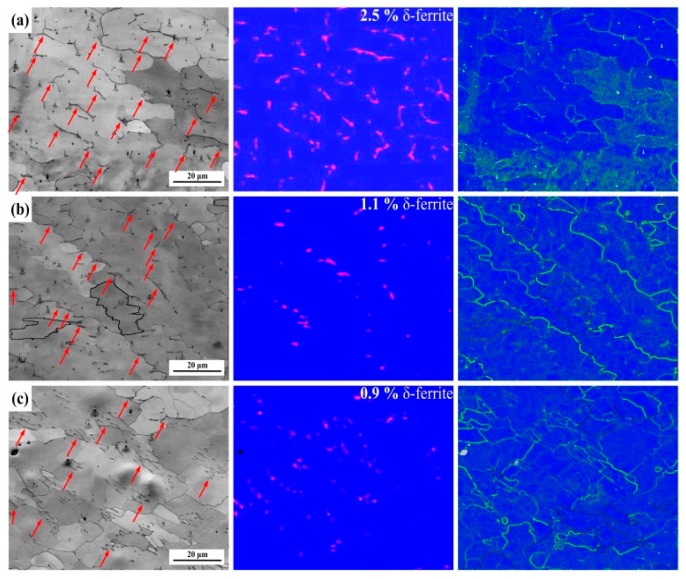
Contrast maps, phase maps, and KAM maps of 1670-14 at different places: (**a**) top, (**b**) middle, and (**c**) bottom.

In order to investigate the local misorientation of δ-ferrite, the weld seam was observed through EBSD in a fine step size of 0.2 μm and a scanning area of 80 × 80 μm^2^. Figure 14 shows the contrast maps, phase maps, and KAM maps of 1670-8 in the weld seam. The microstructures are composed of δ-ferrite and columnar austenite. The δ-ferrite is marked with red arrows in the contrast maps, and the corresponding phase distribution maps show the fractions and distributions of the δ-ferrite (red) and austenite (blue). The KAM maps reveal the relatively higher dislocation density along the δ-ferrite boundaries compared to the austenite boundaries. Thermal cycles of other passes result in the occurrence of recrystallisation and the transformation of δ-ferrite, so the dislocation density also increases from bottom to top. Figure 15 shows the contrast maps, phase maps, and KAM maps of 1670-14 in the weld seam. Similarly, the dislocation density increases from bottom to top, and the boundary of the δ-ferrite has a higher dislocation density than that in grains. The middle and bottom dislocations mainly occur in the boundaries, but the top austenite grains also have a high dislocation density. Compared to the weld seam of 1670-14, the weld seam of 1670-8 contains larger fraction of δ-ferrite due to the low heat input. Nage et al. [32] have found that the mechanical properties increase with the decreasing of δ-ferrite in weld seam.

### 3.4. Mechanical Properties after Welding

**Figure 16:**
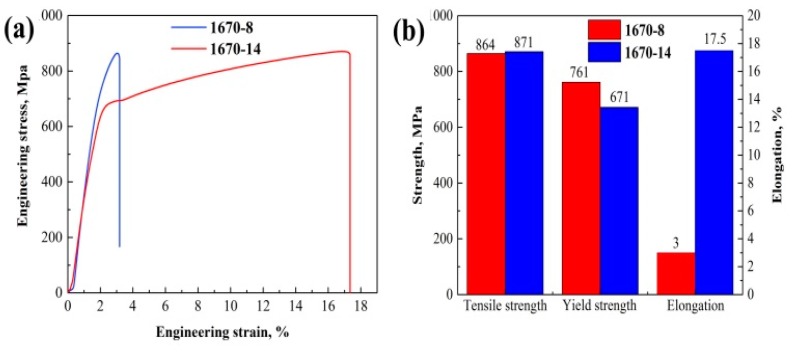
(**a**) stress–strain curve and (**b**) mechanical properties of the different welded joints.

**Figure 17:**
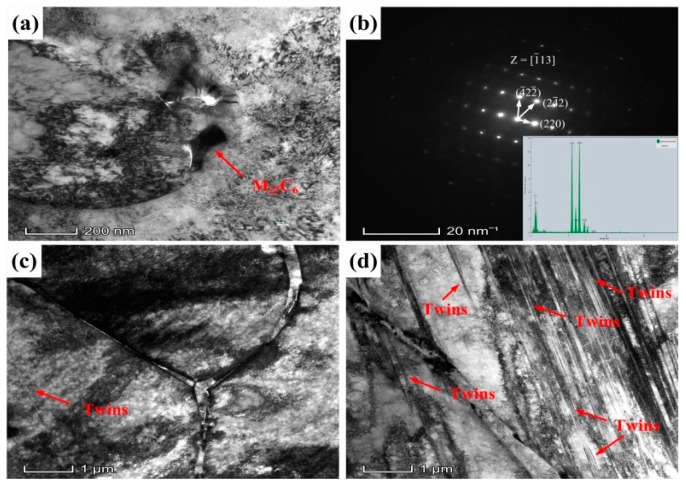
TEM micrographs of the tensile samples of the welded joints: (**a**–**c**) 1670-8, (**d**) 1670-14.

To evaluate the strength and ductility of the welded joints, tensile tests were conducted at 25 °C. A fracture occurred in the weld seam. The tensile stress–strain curves are presented in Figure 16a, and it is observed in Figure 16b that the welded joint of 1670-14 has higher elongation and tensile strength and the difference in tensile strengths of the two welded joints is not significant. As shown in Figure 9, the welded seam of 1670-14 presents a higher dislocation density. It occurs easily for dislocation accumulation and stress concentration. Moreover, the high HAGBs of 1670-8 also represent the preferential crack nucleation sites. On the other hand, Lai et al. [33] reported that cracks initiate preferentially along the austenite/δ-ferrite interface, and high δ-ferrite leads to the elongation loss. After tensile tests, microstructures of welded joints were observed by TEM. Figure 17c,d shows that twins are found in weld seam and 1670-14 has a higher fraction of twins than 1670-8. Twin boundary, as a low energy boundary, has higher strength than random HAGB. Hence, 1670-14 has a higher tensile strength than 1670-8. However, weld seam of 1670-14 has larger grain size for columnar grains after welding under high heat input and cooling rate, which decreases the tensile strength and results in no significant difference in tensile strength. We also found that M_23_C_6_ precipitates along grain boundary of austenite/δ-ferrite in weld seam of 1670-8, and grows into austenite matrix, as shown in Figure 17a. Zheng et al. [34] reported that the ductility of the material reduces with the precipitation of M_23_C_6_. M_23_C_6_ is responsible for the low elongation of 1670-8. 

**Figure 18:**
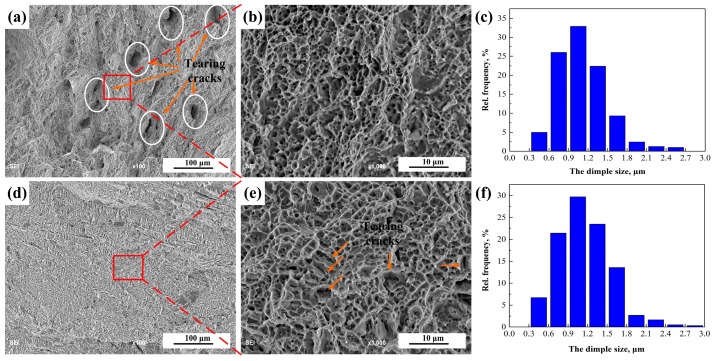
Fracture micrographs and dimple sizes of the tensile samples of the welded joints: (**a**–**c**) 1670-8, (**d**–**f**) 1670-14.

Figure 18 presents the fracture surface of the tensile samples. Figure 18a,d represents the low magnitude fracture morphologies, and Figure 18b,e represent the corresponding amplified morphologies of the red boxes. The typical ductility fracture features with dimples are shown in the fracture surfaces of 1670-8 and 1670-14, but the fracture micrograph of 1670-14 presents relatively flat and homogeneous dimples. Deep and large holes marked in the cycle are formed in the fracture surface of 1670-14. The dimple sizes of fracture surfaces were measured, as shown in Figure 18c,f. The average dimple sizes of 1670-8 and 1670-14 are 1.12 ± 0.38 μm and 1.16 ± 0.38 μm, respectively. Furthermore, fracture morphologies also show that the ductile tearing cracks lead to the damage of 1670-8, while the damage of 1670-14 results from the coalescence of voids. Hence, 1670-14 has better ductility than 1670-8.

During the welding, the thermal cycle of the HAZ can reflect the heat input of the weld seam at every pass, as displayed in Figure 4. The cooling rate reduces with the increase of the number of passes, and 1670-14 presents a higher cooling rate. After the welding, the metals of the weld seam solidify in the primary ferrite mode, then the primary ferrite transforms into austenite. The cooling rate considerably affects the transformation process, and the high cooling rate leads to large primary ferrite. High heat input increases the fraction of recrystallization grains, LAGBs, and the local dislocation density of weld seam, which results in the high tensile strength. However, M_23_C_6_ precipitates in the weld seam of 1670-8 due to the low cooling rate, and reduces the elongation of welded joints. 

## 4. Conclusions

(1) The highly stable welding process and excellently welded joints of the HNASS using a multi-strand composite welding wire were obtained.
(2) After the welding, high heat input and cooling rate results in a noticeable CGHAZ and larger columnar austenite grains. The welded joint obtained under high heat input and cooling rate presented higher fractions of recrystallised grains, LAGBs, KAM; a higher dislocation density; and a higher Schmid factor. Higher dislocation density and Schmid factor indicate the higher tensile strength and the lower yield strength of welded joint, respectively.
(3) The welded joint of the thin plate presents a strong intensity of rotated Goss (G_RD_) ({110}〈1$\bar{1}$0〉) orientation, and the cube (C) ({001}〈100〉) orientation is observed in the welded joint of the thick plate. The S ({123}〈63$\bar{4}$〉) orientation at 65° sections of Euler’s space is weak due to the thermal cycles, which reduces the mechanical properties of welded joints.
(4) Welded joints obtained under high heat input and cooling rate have a lower fraction of δ-ferrite. The KAM maps also revealed a high dislocation density around the primary ferrite, and stress concentration is easy to form along the ferrite.
(5) Welded joint obtained under high heat input and cooling rate has high tensile strength and elongation due to the decrease of δ-ferrite, while low cooling rate leads to the precipitation of M_23_C_6_ in the weld seam; and low heat input also leads to the increasing of δ-ferrite, which decreases the elongation and tensile strength of welded joints.

## Figures and Tables

**Table 1:** Chemical compositions of the base metal, welding wires, and weld seams.

Materials	C	Mn	Cr	Ni	Mo	N	Si	P	S	Fe
HNASS (wt.%)	0.05	18.75	21.12	2.24	0.13	0.68	0.38	0.02	0.01	Bal.
TP-N1670 (wt.%)	0.07	6.62	18.96	8.58	0.01	0	0.85	0.02	0.02	Bal.
1670-8 (wt.%)	0.06	8.78	19.54	7.67	0.08	0.14	0.83	0.02	0.02	Bal.
1670-14 (wt.%)	0.07	8.65	19.38	7.94	0.07	0.14	0.80	0.02	0.02	Bal.

**Table 2:** Welding parameters.

	Pass	Welding Current (A)	Welding Voltage (V)	Welding Speed (mm/min)	Heat Input (KJ/cm)
Thin plate	1	150	16.9	491	3.1
	2	250	25.5	411	9.3
	3	250	25.5	379	10.1
Thick plate	1	160	17.8	335	5.1
	2	250	25.2	356	10.6
	3	250	26.7	356	11.2
	4	250	26.7	356	11.2

## References

[1] Katada Y., Washizu N., Baba H. (2005). Development of high-nitrogen steels in the national institute for materials science. Met. Sci. Heat Treat..

[2] Rasouli D., Kermanpur A., Najafizadeh A. (2019). Developing high-strength, ductile Ni-free Fe-Cr-Mn-C-N stainless steels by interstitial-alloying and thermomechanical processing. J. Mater. Res. Technol..

[3] Kamiya O., Chen Z.W., Kikuchi Y. (2002). Microporosity formation in partially melted zone during welding of high nitrogen austenitic stainless steels. J. Mater. Sci..

[4] Ogawa M., Hiraoka K., Katada Y., Sagara M., Tsukamoto S. (2002). Chromium nitride precipitation behavior in weld heat-affected zone of high nitrogen stainless steel. ISIJ Int..

[5] Zhang H., Zhu W., Zhang T., Guo C., Xu R. (2019). Effect of Brazing Temperature on microstructure and mechanical property of high nitrogen austenitic stainless steel joints brazed with Ni-Cr-P filler. ISIJ Int..

[6] Woo I., Kikuchi Y. (2002). Weldability of high nitrogen stainless steel. ISIJ Int..

[7] Hertzman S. (2001). The influence of nitrogen on microstructure and properties of highly alloyed stainless steel welds. ISIJ Int..

[8] Hosseini V.A., Wessman S., Hurtig K., Karlsson L. (2016). Nitrogen loss and effects on microstructure in multipass TIG welding of a super duplex stainless steel. Mater. Des..

[9] Qiang W., Wang K. (2017). Shielding gas effects on double-sided synchronous autogenous GTA weldability of high nitrogen austenitic stainless steel. J. Mater. Process. Tech..

[10] Park S.H.C., Sato Y.S., Kokawa H. (2007). Microstructure of friction-stir-welded high-nitrogen stainless steel. Mater. Sci. Forum..

[11] Li H.B., Jiang Z.H., Feng H., Zhang S.C., Li L., Han P.D., Misra R.D.K., Li J.Z. (2015). Microstructure, mechanical and corrosion properties of friction stir welded high nitrogen nickel-free austenitic stainless steel. Mater. Des..

[12] Zhang H., Wang D., Xue P., Wu L.H., Ni D.R., Xiao B.L., Ma Z.Y. (2019). Microstructure and corrosion resistance of friction stir welded high nitrogen stainless steel joint. Corrosion.

[13] Mohammed R., Reddy G.M., Rao K.S. (2017). Welding of nickel free high nitrogen stainless steel: Microstructure and mechanical properties. Defence Technol..

[14] Cortes-Cervantes I.S., Lopez-Morelos V.H., Miyashita Y., Garcia-Hernandez R., Ruiz-Marines A., Garcia-Renteria M.A. (2018). Fatigue resistance of AL6XN super-austenitic stainless steel welded with electromagnetic interaction of low intensity during GMAW. Int. J. Adv. Manuf. Technol..

[15] Tolt M.D. (2002). Filler metal selection for welding a high nitrogen stainless steel. J. Mater. Eng. Perform..

[16] Mohammed R., Reddy G.M., Rao K.S. (2016). Effect of filler wire composition on microstructure and pitting corrosion of nickel free high nitrogen stainless steel DTA welds. Trans. Indian Inst. Met..

[17] Zhang H.T., Chang Q., Liu J.H., Lu H., Wu H., Feng J.C. (2014). A novel rotating wire GMAW process to change fusion zone shape and microstructure of mild steel. Mater. Lett..

[18] Randle V. (2009). Electron backscatter diffraction: Strategies for reliable data acquisition and processing. Mater. Charact..

[19] Zhang W., Wang X., Hu Y., Wang S. (2018). Quantitative studies of machining-induced microstructure alteration and plastic deformation in AISI 316 stainless steel using EBSD. J. Mater. Eng. Perform..

[20] Roach M.D., Wright S.I., Lemons J.E., Zardiackas L.D. (2013). An EBSD based comparison of the fatigue crack initiation mechanisms of nickel and nitrogen-stabilized cold-worked austenitic stainless steels. Mater. Sci. Eng. A.

[21] Lu J., Wu X., Liu Z., Chen X., Xu B., Wu Z., Ruan S. (2016). Microstructure and mechanical properties of ultrafine-grained copper produced using intermittent ultrasonic-assisted equal-channel angular pressing. Metall. Mater. Trans. A..

[22] Mccabe R.J., Richards A.W., Clarke K.D., Beyerlein I.J., Knezevic M. (2015). Microstructure effects on the recrystallization of low-symmetry alpha-uranium. J. Nucl. Mater..

[23] Kamaya M., Fonseca J.Q.D., Li L.M., Preuss M. (2007). Local plastic strain measurement by EBSD. Appl. Mech. Mater..

[24] Xu Y., Nie Y., Wang M., Li W., Jin X. (2017). The effect of microstructure evolution on the mechanical properties of martensite ferrite steel during long-term aging. Acta Mater..

[25] Gussev M.N., Field K.G., Busby J.T. (2015). Deformation localization and dislocation channel dynamics in neutron-irradiated austenitic stainless steels. J. Nucl. Mater..

[26] Ganesan V., Mathew M.D., Sankara R.K.B. (2013). Influence of nitrogen on tensile properties of 316LN SS. Mater. Sci. Technol..

[27] Hammar O., Svensson U. (1979). Influence of Steel Composition on Segregation and Microstructure During Solidification of Austenitic Stainless Steels. Solidification and Casting of Metals.

[28] Vasudevan M., Bhaduri A.K., Raj B., Rao K.P. (2007). Artificial neural network modeling of solidification mode in austenitic stainless steel welds. Mater. Sci. Technol..

[29] Bhaduri A.K., Srinivasan G., Klenk A., Raj B. (2009). Study of hot cracking behaviour of 14Cr-15Ni-2.5Mo Ti-modified fully austenitic stainless steel using varestraint and hot ductility tests. Weld. World.

[30] Lo K.H., Shek C.H., Lai J.K.L. (2009). Recent developments in stainless steels. Mater. Sci. Eng. R.

[31] Jahanzeb N., Shin J.H., Singh J., Heo Y.U., Choi S.H. (2017). Effect of microstructure on the hardness heterogeneity of dissimilar metal joints between 316L stainless steel and SS400 steel. Mater. Sci. Eng. A.

[32] Nage D.D., Raja V.S., Raman R. (2006). Effect of nitrogen addition on the microstructure and mechanical behavior of 317L and 904L austenitic stainless steel welds. J. Mater. Sci..

[33] Lai C.L., Lu W.F., Huang J.Y. (2014). Effect of δ-ferrite content on the stress corrosion cracking behavior of cast austenitic stainless steel in high-temperature water environment. Corrosion.

[34] Zheng L.G., Hu X.Q., Kang X.H., Li D.Z. (2015). Precipitation of M_23_C_6_ and its effect on tensile properties of 0.3C-20Cr-11Mn-1Mo-0.35N steel. Mater. Des..

